# Editorial: Neuroendocrine Control of Energy Homeostasis in Non-mammalian Vertebrates and Invertebrates

**DOI:** 10.3389/fendo.2020.00404

**Published:** 2020-06-19

**Authors:** Suraj Unniappan, Ian Orchard, Maria Jesus Delgado

**Affiliations:** ^1^Laboratory of Integrative Neuroendocrinology, Department of Veterinary Biomedical Sciences, Western College of Veterinary Medicine, University of Saskatchewan, Saskatoon, SK, Canada; ^2^Department of Biology, University of Toronto Mississauga, Mississauga, ON, Canada; ^3^Department of Genetics, Physiology and Microbiology, Complutense University of Madrid, Madrid, Spain

**Keywords:** food intake, fish, energy homeostasis, invertebrates, metabolism, hormones

Energy is critical for all physiological processes, including the growth and reproduction of animals, and so the maintenance of energy balance is essential for species survival. Imbalances in energy intake and expenditure lead to abnormal physiology and debilitating diseases, contributing to a growing interest in the study of energy balance. Among the myriad of factors that contribute to energy intake and expenditure, hormones and endogenous factors with hormone-like actions are prime elements. The use of comparative approaches to study hormones regulating metabolism and other physiological functions are very important and impactful. It helps to address species-specific differences in hormones and their action, which are essential in tailoring custom approaches to utilize endocrinology for the benefit of species survival, sustainability, and health. Comparative neuroendocrinology research using fish (aquaculture and food sustainability) and invertebrates (disease and/or vector control) are examples of this. Both mammals and non-mammals, and invertebrates have been used to study the mechanism of endocrine and metabolic diseases. Several hormone-based pharmaceuticals that are commercialized for metabolic and endocrine diseases have its roots in comparative endocrinology research using model organisms. Due to these, we conceived and coordinated the publication of a Research Topic on the “*Neuroendocrine Control of Energy Homeostasis in Non-mammalian Vertebrates and Invertebrates.”* There were 14 original research articles and 4 reviews in this issue, covering a broad spectrum of research across both invertebrates (8 articles) and vertebrates (10 articles). This e-book presents the collection of the above articles that highlight many recent advances in the neuroendocrine regulation of energy homeostasis. The research presented here encompasses cellular, molecular, and organismal level biology, employing a large number of cutting edge tools. These include techniques associated with genetic and molecular biology, biochemistry, behavior, physiology, and pharmacology; the reports are state-of-the art and timely in nature.

Four review articles consider the biology of insulin-like peptides (ILPs) signaling in mosquitoes, neuropeptide F (NPF) in invertebrates, the roles of ghrelin and motilin in regulating gut motility in vertebrates, and the gut microbiota and energy homeostasis in fish. Sharma et al. provide a very comprehensive commentary on the structure, distribution and functions of ILPs in regulating metabolism, growth and reproduction, as well as the vectorial capabilities of mosquitoes by influencing mosquito-pathogen interaction. Overall, this review highlights the significance of ILPs in an important disease vector, and points the reader to priorities that researchers should focus on in future research. Fadda et al. summarize the roles of the neuropeptide Y (NPY) family, neuropeptide F (NPF) peptides, in the regulation of energy balance in invertebrates. Their very systematic review provides an excellent overview of NPF history, nomenclature, and signaling, and the roles of NPF in energy homeostasis in an array of invertebrate groups. Meanwhile, Kitazawa and Kaiya enlighten us on the roles of motilin and ghrelin, two very important gut peptides, on gut motility in vertebrates. This very thorough review covers a large number of species from teleost fish to humans, studying the motility effects of these two gut peptides. While both peptides and their receptors are present across most species studied, there appears to be species-specific differences in their roles on gastrointestinal motility. In addition, huge gaps in knowledge still exist when it comes to a full understanding of these two peptides. Butt and Volkoff focus on a very different aspect; the role of gut microbiota on metabolic homeostasis, which remains a current area of prolific research focus in vertebrates. This article discusses the role of gut microbiota in regulating energy balance and energy-utilizing functions including stress, reproduction, immunity, development and growth. It considers the factors affecting gut microbiota, which include age, sex, genetics, life stage, diet and nutrient composition, and how the gut microbiota respond to the use of antibiotics, pre-/pro- and symbiotics. Overall, the knowledge covered under these reviews has implications in vector control, and aquaculture (fish metabolism).

Among the original research articles, six focus on invertebrates. Post et al. report their interesting findings on how ILPs regulate longevity by deactivating glycogen phosphorylase, which is a key player in glycogen storage and gluconeogenesis. Defferari et al. report the first insulin receptor, and showed how conserved the insulin cell signaling mechanisms that contribute to energy homeostasis are in a blood feeding insect, *Rhodnius prolixus*. Kawabe et al. study bombyxin II, an ILP in *Bombyx mori*. Their key findings indicate that bombyxin II injection resulted in an increase in oxygen consumption and an upregulation of an enzyme activator of glycolysis in a tissue specific-manner. Meanwhile, Marco and Gäde present an original article that focuses on the ligand-receptor interactions of five forms of the adipokinetic hormone (AKH) found in the sphingid moth, *Hippotion eson*. They found that there is no signature bioactive core in these peptides, and the conformation provided by the full-length active peptide is ideal for most effective receptor binding. Sharma, Pooraiiouby et al. in a second article (original research), furnish details of the of ILPs in black-legged tick, *Ixodes scapularis*. Their initial characterization found ILP sequences, and their tissue-, and life-/feeding-stage-specific expression in this tick, and provided impetus for future research on these important metabolic peptides in ticks. Wahedi et al. add new information on structure-activity relationships of *Aedes aegypti* receptors for insect hormones with gonadotropin releasing hormone-like properties, namely AKH, corazonin (CRZ) and AKH/CRZ-related-peptide. They identified a number of critically-positioned amino acids within these peptides that enable receptor binding. All these articles contribute significantly to our understanding of cellular and/or organismal metabolic processes and the mechanism by which hormones regulate such processes in a variety of invertebrates.

Among articles that inform us on new research data from vertebrates, most used fish as a model organism. Perelló-Amorós et al. report the nutritional regulation of ghrelin and its receptors in a cultured fish, gilthead sea bream *(Sparus aurata)*. They found that the tissue-specific expression of endogenous ghrelin, an orexigen, and its receptors fluctuate during fasting. While Perelló-Amorós et al. provide new insights on an orexigen Blanco et al. pursue nesfatin-1, an anorexigen in another cultured fish, the rainbow trout *(Oncorhynchus mykiss)*. They found that injection of nesfatin-1 into the brain resulted in a suppression of feeding, an increase in glucose sensing and in lipogenic enzymes in the brain. Meanwhile, the same treatment reduced gluconeogenic and lipogenic mRNAs, and enhanced enzymes that promote fatty acid oxidation. Chen et al. look into how temperature affects feeding in goldfish *(Carassius auratus)*. They found a decrease in feeding with lowering temperatures and corresponding changes in appetite regulatory peptides. Liu et al. focus on the melanocortin 4 receptor (Mc4r) and its role in the growth of medaka *(Oryzias latipes)*. While the growth of medaka that had genetically lost Mc4r was significantly suppressed compared to wildtype controls, no changes in puberty were found in the *mc4r* knockout fish. Nakamachi et al. indicate that adenylate cyclase-activating polypeptide (PACAP) and PAC1R are involved in the regulation of feeding in zebrafish *(Danio rerio)*, where intracerebroventricular injection of PACAP reduced feeding without affecting locomotor activity. Otero-Rodiño et al. use morphometric analysis to map the glucosensing system within the rainbow trout brain. They found glucokinase expressed in neurons that co-expressed an orexigen, agouti-related peptide, in the rostral hypothalamic neurons of the trout brain. Gómez-Boronat et al. reveal the diurnal changes in n-acylethanolamines (NAEs), a group of lipid signaling molecules in goldfish. NAEs showed a daily rhythm in their tissue specific expression, and were significantly elevated post-prandially in the gut. This suggests an anorectic role for NAEs in goldfish. These articles provide novel insights on the hormonal regulation of energy balance in fish.

The sole article on avians reports primary research on the neurosecretory protein GL (NPGL). Shikano et al. reveal that the chronic intracerebroventricular infusion of NPGL resulted in a depot-specific increase in white adipose tissue mass, which indicates a role for this peptide in creating a positive energy balance. They also found an increase in liver mass, suggesting a positive role for centrally administered NPGL on *de novo* lipogenesis in chicken. There were no submissions on studies using mammals.

In conclusion, this e-book provides research data and reviews that furnish opportunities to gather significant new knowledge on hormones and metabolism. From neuroanatomy to cell physiology to whole animal biology, the authors consider several key determinants that influence hormones and their actions. These include sex, age, temperature, locomotion, feeding, tissue-specific expression, structure of hormones, receptors, mode of administration and species used. The extensive nature of the research articles assisted in the broader scope of this e-book. We tremendously enjoyed working with the authors, reviewers, and editorial staff, whose contributions were immeasurable to the success of this Research Topic and the resulting e-book. As co-editors, we are deeply thankful to everyone involved in making this issue a success. It is our sincere hope that this compendium will remain useful to researchers interested in the neuroendocrinology of energy homeostasis. Finally, we want to thank our readers, who are also the ultimate reviewers of the work presented. Enjoy reading!

## Author Contributions

SU prepared the manuscript and revised and finalized for submission. IO and MD edited the manuscript. All authors contributed to the article and approved the submitted version and author's page proofs.

## Conflict of Interest

The authors declare that the research was conducted in the absence of any commercial or financial relationships that could be construed as a potential conflict of interest.

